# Shannon Entropy for Measuring Spatial Complexity Associated with Mean Annual Runoff of Tertiary Catchments of the Middle Vaal Basin in South Africa

**DOI:** 10.3390/e21040366

**Published:** 2019-04-04

**Authors:** Masengo Ilunga

**Affiliations:** Department of Civil and Chemical Engineering, College of Science, Engineering and Technology, Private Bag X6, Florida Campus, University of South Africa, Florida, 1710 Johannesburg, South Africa; ilungm@unisa.ac.za; Tel.: +27-11-471-2791

**Keywords:** Shannon entropy, complexity, mean annual runoff, water resource, tertiary catchment, iso-information redundancy, iso-information transmission

## Abstract

This study evaluates essentially mean annual runoff (MAR) information gain/loss for tertiary catchments (TCs) in the Middle Vaal basin. Data sets from surface water resources (WR) of South Africa 1990 (WR90), 2005 (WR2005) and 2012 (WR2012) referred in this study as hydrological phases, are used in this evaluation. The spatial complexity level or information redundancy associated with MAR of TCs is derived as well as the relative change in entropy of TCs between hydrological phases. Redundancy and relative change in entropy are shown to coincide under specific conditions. Finally, the spatial distributions of MAR iso-information transmission (i.e., gain or loss) and MAR iso-information redundancy are established for the Middle Vaal basin.

## 1. Introduction

Hydrological systems are characterised with a level of uncertainty [1,2], dispersion or compactness [3,4], uniformity or concentration [5]. For example, higher entropy is associated with higher dispersion [3,4]. From information theory perspective, Shannon entropy is the average uncertainty of a random variable and gives on average the minimum number of bits needed, to characterise the random variable [6]. In other words, entropy is the expected value of a random variable called information and is based in the event’s probability [1,2]. The expected surprise about the truth can be an interpretation of entropy-measure for uncertainty [7] or ignorance [8]. Since entropy relates to measuring uncertainty, information relates to measuring the certainty of a system. A system generally moves to a less orderly phase from an orderly phase and from less probable phase to more probable phase, in this way entropy is maximized and a probability set defines the entropy magnitude [9]. Less credible predictions about a system are usually associated with higher entropy [2].

Entropy is a measure of complexity [10], hence high entropy relates to high complexity and relates to more information [11,12] of hydrological systems. Unpredictable, unstructured and complex systems can be described by information entropy [12]. The distribution of water resources over a basin is associated with entropy and usually present some dispersed configuration [5,13].

Since a basin is a complex hydrological system due to the interactions of variables such as streamflow, evaporation, rainfall, system physical features, etc. [14], these variables are associated with spatial and temporal information over the basin. Runoff (e.g., mean annual runoff (MAR)) on its own has been considered as the result of a complex combination of precipitation, land use/change, evaporation, physical catchment characteristics, etc. and plays an immense role in the planning and operation of water resources [15,16]. Hence the “variability” of representative observed data, i.e., streamflow, reflects hydrological system “complexity” [14]. Hydrological adaptive complex systems are generally characterised by diversity, connectedness, dispersion, concentrating, resilience and resistance [17] and could be basins for instance. Entropy theory was shown to be a good tool to understand water and land resources as adaptive complex systems, i.e., involving many units with multidimensional functions and benefits [18]. These units are defined at three levels: system-level agents, sector-level agents and user-level agents [18].

There is no doubt Shannon entropy has been used widely in hydrology and water resources, specifically for measuring information content of variables and information transmission (i.e., gain or loss) among variables [19,20]. These variables characterise the hydrological system. The reduction of uncertainty of a hydrological system is indirectly the gain of information of the system [1,20]. Conversely, the increase of uncertainty is indirectly a measure of the loss of information of the hydrological system. This shows the equivalence between information and the reduction of uncertainty [6]. Hence an information gain or loss can express the difference between two uncertainty values [8]. In the perspective of Shannon entropy, three main aspects of hydrological information gain or loss could be considered:

- Information transfer/transmission among hydrological variables, which belong to different sites, e.g., rainfall stations or streamflow gauges. This is usually done via models [1,20,21]. For instance, information transfer among rainfall data sets, was defined spatially and temporally, via univariate and multivariate models [22,23] and streamflow modelling [24]. Information gain or loss is defined by the informational correlation or transferrable information between observed and simulated streamflow (rainfall). The directional information transfer index (DIT) was derived as a measure of information transfer between two given hydrological stations [21] and normally varies between 0 and 1. Generally, the DIT decreases for stations situated far away from the central stations [4]. There is information gain from the sending station to the receiving station and information loss for the opposite. The transferable information is referred to as transinformation, e.g., [25,26], mutual or overlapped information [26] and shared or redundant information [27]. In this way, it refers to common information among variables [28]. Information redundancy was shown to be very important in rainfall network optimization, i.e., the first rainfall site selected has the highest entropy and the last one having the least redundancy in a way to reduce uncertainty in the network [27,29]. This implies that the first station with the minimum overlapped information is added first to the station having highest entropy [26]. The ultimate goal is to maximise information, hence minimise redundancy [30]. Redundancy relates to repetitive information, which does not bring essentially new knowledge about the system. Other than information theory, information redundancy can be determined from a set of hydrologic statistics such as K-means, standard deviation, self-organising map (SOM) clustering to categorise flow regimes [31], which are not covered in the scope of this study.
- Information gain can be understood in the like context of Kullback Leibler (KL) divergence, which characterises the classic information difference [11]. It is a measure of the relative entropy when comparison is carried out on two distributions; one is assumed close to the true uncertainty and the other is an estimated uncertainty [7]. In the context of KL information, the distance between two probability distributions can be measured by the relative entropy [32]. For the classic information difference that will apply to this study, KL defines the proportion of information that a hydrological system would require to adjust itself to the maximum state of uncertainty [11]. In this sense, KL is related to the relative entropy as a measure of complexity [12,19]. Spatial variation of relative entropy was found to be a good measure of the complexity of streamflows since the results obtained were consistently produced and were independent of the magnitude of the data [11]. KL is known as the complexity ratio called also redundancy [11]. Low values of complexity ratio are associated with complex systems (high uncertainty) while its high values are associated with low complex systems (low uncertainty). Redundancy (*R*) varies between 0 and 1, which correspond to a system of high uncertainty and low uncertainty respectively. These 2 values relate to complete unknown information (i.e., high ignorance) and known information (i.e., low ignorance) about a hydrological system.
- The relative change in entropy (RCE) of a hydrological system could be defined from one phase to the other and these phases occur in different periods of time. Information gain/loss between sets of data [13] was implicitly approached as the relative change of marginal entropy between two phases. In essence, the difference in entropies between two consecutive phases [3] is compared with the entropy of the initial phase.

The current study is specifically aligned with the last two aspects of information gain or loss. Firstly, to account for the information transmission associated with MAR of tertiary catchment (TCs) of the Middle Vaal basin, RCE is evaluated between consecutive phases. In hydrology of South Africa, a TC is divided into quaternary catchments (QCs) which are the basis for the estimation of MAR in water resources management. A quaternary catchment is the foundational areal unit of the river basin and it is considered preferable to vary the catchment area according to runoff, i.e., the bigger the runoff volume, the smaller the QC. TC is a catchment formed by sub-catchments, i.e., QCs sharing similar hydrological characteristics. In a similar way, a specific group of TCs yields a secondary catchment. It implies that the primary catchment is composed of a group of secondary catchments with similar hydrological properties. The nomenclature of a QC has 4 characters. The first and last characters are letters; the intermediate characters are numeric values. For example in the quaternary catchment C23A, C is the primary catchment, in this case the Vaal region, 2 relates to the secondary catchment, 3 is the tertiary catchment and A characterises the quaternary catchment. This nomenclature was initiated by the Department of Water and Sanitation (DWS) as well as the Water Research Commission (WRC) of South Africa, which operate under the South African government. In South Africa, water researchers, consulting firms and other entities have adopted this nomenclature.

The Lower Vaal together with the Upper Vaal and Middle Vaal basins forms the current Vaal management area. Like [13,15], surface water resources (WR90) of South Africa 1990 (WR90), 2005 (WR2005) and 2012 (WR2012) data sets were used for MAR, specifically for the Middle Vaal. WR90, WR2005 and WR2012 data sets were considered in this study as the different phases of the water resources of South Africa. It should be noted that, in the history of hydrological appraisals of South Africa, studies are commissioned by WRC and the first comprehensive data set in its kind was WR90. This data set was commissioned in 1990 and completed in 1994. It provided valuable data and information for water resources planning and development [33], since then it has been adopted by users from different categories, such as students, researchers, engineering practitioners and consultants, government institutions, and water industry users [16]. In a period of 15 years later, another study of surface water resource appraisal was commissioned to update the first data set and yielded the WR2005, which was completed in 2011. In 2012, WRC initiated an update of WR2005 to give birth to WR2012, which was finalised in 2016. So far WR90, W2005 and WR2012 are the well documented hydrological phases and are freely ready available from WRC. Updating hydrological data is common since data and information impact on the decision making for the operation, planning and management of water resources. The MAR spatial distribution impacts on water resources management and development. Secondly, information redundancy is evaluated to establish the necessary information contained in each phase. The study also established a relationship between RCE and *R*, under specific conditions. Finally, a spatial distribution of information redundancy associated with MAR was also derived for the Middle Vaal basin, i.e., areas of the same redundancy called iso-information redundancy could be identified. The spatial distributions of information transmission derived specifically from RCE were also established. In this way, iso-information transmission zones can be determined.

It should be noted that in the old configuration of 19 water management areas (WMAs) of South Africa, Middle Vaal was categorised as a WMA [16,34], which is essentially a basin. The old configuration was based on practical considerations (hydrological, economical, etc.), unlike the new configuration which has nine WMAs was determined on a bias of political considerations. Moreover, no publication of surface water resources exists yet with reference to this new configuration. WR90, WR2005 and WR2012 data sets were published within the context of 19 WMAs and the same data are currently used. Hence the new configuration does not impact on hydrological data in the Middle Vaal basin. This study will use the word “basin” more than “WMA”, with respect to Middle Vaal. The words complexity ratio and information redundancy will be used interchangeably. Often the word information before ”gain” or “loss” will be omitted to mean information gain or loss. Change between WR90 and W2005 phases will be written as WR90-W2005. The same applies to change between WR2005 and WR2012. Information transmission will be understood as loss or gain of information, specifically when referring to the relative change in entropy between phases. Information transfer and information transmission will mean the same.

The rest of the paper is organised as follows: Section 2 presents an overview on the Middle Vaal basin and data used in the study. Section 3 deals with the methodology used to determine ultimately the spatial configuration of information transmission among phases and information redundancy contained in each phase. The complexity measure of hydrological systems from a theoretic information aspect is critical in this section. The equivalence between *R* and RCE is also derived. Section 4 gives results and discussion of the application of the methodology on Middle Vaal basin. Conclusions and suggestions are presented in Section 5.

## 2. Study Area and Data Availability

**Figure 1:**
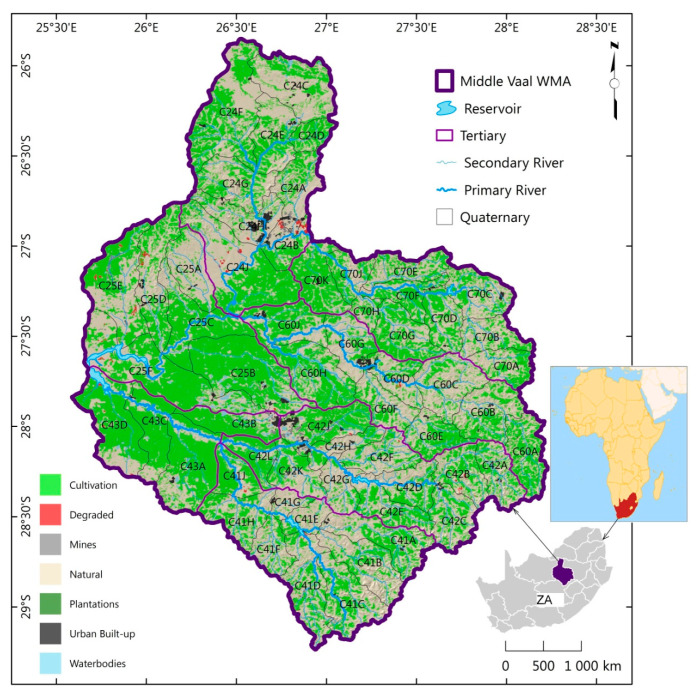
Tertiary catchments comprising quaternary catchments in the Middle Vaal basin of South Africa (ZA) shown at the left. The African map has been extracted from Aquastat [35], as presented at the right.

The Middle Vaal basin is located in South Africa, specifically between the Upper Vaal and Lower Vaal basins. It shares borders with the Crocodile (West) and Marico and the Upper Orange basins [34]. The Vaal River plays a major role as the main river in the Vaal region (Upper, Middle and Lower basins), which is the economic hub of Gauteng Province and of South Africa at large. Figure 1 shows the location of Middle Vaal basin, which is between 25°30’ and 28°30’ E longitudes and between 25°30’ and 29°30’ S latitudes. The map of Africa in Figure 1 has been added to give a context of the study area in the continent. Noticeable spatial fluctuations in climatic conditions, water availability, economic development and population distribution charasterise the Middle Vaal water management area.

To strategise the water resource management, three sub-areas were considered in the Middle Vaal [34], i.e., the Rhenoster-Vals sub-area comprising TCs C24 and C25, the Middle Vaal sub-area formed by C60 and C70 and the Sand-Vet sub-area covering C41, C42 and C43.

Grassland is the main vegetation. Middle Vaal basin has relatively a flat topographic feature with the occurrence of hilly feature in the South-East. The geologic formation is largely dolomite from Orkney towards the northern area of the basin. Mainly gold mining around Welkom and Klerksdorp, agriculture and trade are the economic activities. The basin contributes 4% approximately to the gross domestic product (GDP) of South Africa. The basin has a temperate climate, which is generally semi-arid since South Africa is a semi-arid country with its mean annual rainfall of 400 mm generally below the world average mean annual rainfall. Due to aridity, a high proportion of water is lost through evaporation due to excessive temperatures experienced in summer for most regions of the country [34,36]. In Middle Vaal, precipitation is generally in the form of rainfall, which occurs in summer time, i.e., from October to December, with December and January as the hottest months. In January alone, the highest Class A-pan evaporation varies monthly between 180 mm to 260 mm, whereas the month of June experiences the lowest evaporation between 80 mm and 110 mm [36]. The mean annual precipitation (MAP) ranges from 700 mm to 400 mm in the South-East and in the West of Middle Vaal respectively. There is occurrence of thunder storms during summer rainy season. The mean annual evaporation (MAE) is approximately 1900 mm, which is greater than the MAP [34]. Hence there is evidence from the different studies led by WRC and compiled in the form of data sets, i.e., WR90, WR2005 and WR2012 that the mean annual evaporation is well above the mean annual rainfall [37]. This situation led South Africa in the past to build more water storage capacities, in particular hydraulic structures such as dams and to establish water transfer schemes among basins in a way to balance water demand and supply.

Data for tertiary catchments were extracted from WR2012 reports [37] and are displayed in Table 1. Details of data on QCs can be obtained from these reports, which are the recent publications for water resources of South Africa and include the previous ones, i.e., WR90 and WR2005. In principle hydrological data from 1920 to 1989, 1920 to 2004 and 1920 to 2012 are included in WR90, WR2005 and WR2012 respectively. In Table 1, the values of MAE and MAP are based on WR2012. It is observed that despite similar TC areas, the huge difference in MAR between C24 and C25 can be due to catchment characteristics other than area. Such characteristics are, for instance, geology, soil texture, topography, vegetation, etc. The topography in C25 is predominantly flat as compared with C24 [38], with soil depths generally moderate in the Middle Vaal basin. From the geological formation, there are more sedimentary and exclusive rocks in C24 than in C25 [38]. For instance the topography of C24 is relatively higher than that of C25 and can favor more runoff in C24. The geology of C24 may lead to lower infiltration rate and higher runoff as opposed to C25. The combined effect of the abovementioned characteristics may contribute to the higher net MAR for C24 as opposed to C25. The data from WR90 show clearly that the dimensionless parameter related to the reduction in runoff volume events is lower in C24 than in C25 [39]. In this case, the parameter for C24 is approximately half of that of C25 and expresses also the average infiltration rate [39], which should be higher for C25 than for C24. A similar observation can be made between C42 and C43, in terms of their MAR.

## 3. Methods

In this section, entropy is explored as a measure of complexity of hydrological systems.

### 3.1. Relative Entropy 

In its discrete form, the Shannon marginal entropy index $S{\left(X\right)}_{j}$ of a hydrological variable *X* (e.g., runoff) in the *j*-th TC catchment (*j* = 1, …, *q*) is determined by Equation (1):

(1) $$S{\left(X\right)}_{j}=\overset{k}{\underset{i=1}{\sum }}S\left(x_{i}\right)$$

where *q* is the number of TCs in a basin and $S\left(x_{i}\right)$ is the marginal entropy of the event *x_i_* and is given by Equation (2) below:

(2) $$S\left(x_{i}\right)=-\frac{x_{i}}{X_{T}}\log\frac{x_{i}}{X_{T}}$$

The variable *X* takes on event value *x_i_* (*i* = 1, 2, 3, …, *k*) and the total value of events is *X_T_*; i.e., $X_{T}=\sum_{i=1}^{k}x_{i}$. The value *x_i_* can be MAR for a specific *i*-th QC in a given TC of a basin, i.e., the Middle Vaal. The MAR of a TC is made of the MAR contribution from each QC. The base of the logarithm is the unit of *S*(*x_j_*). The unit is in bits, in Napiers, and in decibels (dB) for bases 2, *e* and 10 respectively. Equations (1) and (2) show that entropy can be bound generally between 0 and $S_{j,max}$ (maximum entropy in the *j*-th TC). $S_{j,max}$ corresponds to the equi-probable outcomes of the hydrological variable and is equal to logN (N is the number of QCs in the *j*-th TC). The distribution of values closer to 0 is compact as opposed to values closer to $S_{j,max}$ showing an evenly dispersed or spread distribution [3].

Equation (1) satisfies the following condition:

(3) $$\overset{k}{\underset{i=1}{\sum }}\frac{x_{i}}{X_{T}}=1$$

The relative entropy is the ratio between uncertainty and maximum entropy [1,11,12,19], as defined in Equation (4) with respect to the *j*-th TC:

(4) $$r_{j}=\frac{S_{j}}{S_{j,max}}$$

The bigger the ratio, the higher the uncertainty of a system. Hence hydrological systems having entropy close to the maximum entropy will display higher uncertainty that characterises higher dispersion of the variable distribution. Relative entropy is sometime called information discrimination or information divergence between two distributions and relates to information increase, or decrease [6]. In the relative entropy expression, the maximum entropy is a normalising factor for marginal entropy [10,11]. For this study, relative entropy is defined for a given phase; however it makes a comparison between the actual entropy and the maximum entropy of the phase [40,41]. In principle, entropy assessment of complex systems should involve more than one variable.

### 3.2. Complexity Ratio or Information Redundancy

Redundancy consists in repeating the signal that does not generate new information [42] for a given system. That is to say, redundant information does not change the usefulness of the existing information contained in the system. However, it is cautioned that redundant information may be helpful to detect errors in the system and possibly correct them [43]. Redundancy is generally considered to be the complement of relative entropy and shows the complexity of a system [11], i.e., one minus relative entropy defines information theoretic redundancy [43]. The redundancy *R_j_* of the *j*-th TC in a basin is given by Equation (5) below:

(5) $$R_{j}=\frac{S_{j,max}-S_{j}}{S_{j,max}}$$

(6) $$R_{j}=1-r_{j}$$

where $r_{j}$ is the relative entropy for *j*-th TC and the remaining terms have been defined previously.

The more complex is the hydrological system, the lower the redundancy and vice versa. Referring to [11,42], complex hydrological systems could be of interest since they contain useful information and minimum redundancy. Otherwise the information in the system is relatively low. This is a consequence of the principle of maximum entropy. Like relative entropy, the upper bound in the information redundancy equation is the maximum entropy, which is the common denominator for both relative entropy and redundancy. It can be viewed as a normalised or standardized indicator of the information gain [11]. Thus, like relative entropy, redundancy is defined for a given phase (at a given time); however it makes a comparison between the information gain and the maximum entropy. Since it is almost impossible to have idealised systems (i.e., error free), information redundancy will always be part of any system; i.e., it can be part of the system without losing information of the system or it can be deleted in a structured way without loss of information [43].

### 3.3. Relative Change in Entropy (RCE) as a Measure of Information Gain (Loss)

RCE differs essentially from relative entropy and redundancy in the sense that it is defined between 2 hydrological phases occurring respectively at different times. The concept was used by [13,15] and a similar concept was used by [3]. RCE shows the uncertainty change involved when a system moves between phases 1 and 2. Mathematically, the expression of RCE($\Delta_{j,2-1}$) for the *j*-th TC that moves from 1 (initial phase) to 2 (final phase) is given by Equation (7):

(7) $$\Delta_{j,2-1}=\left[\frac{S_{j,2}-S_{j,1}}{S_{j,1}}\right]\times 100$$

Unlike *R* and *r* which are always positive, RCE can be positive or negative depends on the uncertainty levels that characterise the two phases. Positive and negative changes in RCE correspond to loss of information and gain of information respectively. Moreover, the common denominator in the expression of RCE is not necessarily the maximum entropy, unlike for *R* and *r*. The rest of Section 3 gives the steps undertaken to compute relative entropy, redundancy, relative change in entropy and to determine the maps related to iso-information transmission/redundancy:

### 3.4. Determination of Relative Entropy and Information Redundancy

For a particular phase, relative entropy and information redundancy of a hydrological system were computed by Equations (4) and (6), respectively. Based on its function on water resources sustainability and resilience [13,15], hydrological system (phase) was mainly characterised by MAR. When *r* approaches a value of 0, the spatial distribution of MAR will be concentrated in one TC or few TCs. When *r* approaches 1, the MAR spatial distribution will be dispersed evenly across the TCs in the Middle Vaal basin.

On the contrary, the information redundancy, as the complementary part of relative entropy, will take 1 and 0 values respectively. Low *R* will have less repetition in the spatial configuration of MAR, and will not necessarily bring new information about the hydrological system. *R* and *r* are approached in this study as intraphase information parameters, derived from their classic determination as defined earlier.

### 3.5. Determination of Relative Change in Entropy between Phases

The relative change in entropy between two consecutives phases 1 and 2 was computed by using Equation (7). In the specific case of Middle Vaal, the expressions of RCE for WR90-WR2005 and WR90-WR2005 in the *j*-th TC, were given by Equations (8) and (9), respectively:

(8) $$\Delta_{j,WR2005-WR90}=\left[\frac{S_{j,\left(WR2005\right)}-S_{j,\left(WR90\right)}}{S_{j,\left(WR90\right)}}\right]\times 100$$

(9) $$\Delta_{j,WR2012-WR2005}=\left[\frac{S_{j,\left(WR2012\right)}-S_{j,\left(WR2005\right)}}{S_{j,\left(WR2005\right)}}\right]\times 100$$

Normally smaller values of RCE implied that the distribution of the yield of water resources remained almost unchanged when the basin changed from phase 1 to phase 2. Since a methodology for the determination of thresholds values for the 2 entities, i.e., *R* and RCE is almost inexistent in the literature, values less than 10% were arbitrarily considered to imply that these entities remain almost constant. In the context of this study, RCE is an interphase information parameter unlike *R* and *r*.

### 3.6. Determination of Equivalence between RCE and R

When the initial phase approaches the maximum entropy, it can be shown that information loss calculated from RCE between 2 successive phases can be simply determined from the information redundancy of either phase 1 or 2 and vice-versa. Hence, at the limit when the initial phase is characterised with higher entropy, e.g., close to the maximum entropy, Equation (10) can be obtained as follows:

(10) $$\left[\frac{S_{j,2}-S_{j,1}}{S_{j,1}}\right]=-R$$

Equation (10) can be viewed as a special case that establishes the equivalence between information transmission and information redundancy. Strictly speaking, there is equivalence only when the absolute value of information transmission between phases is considered. The above equation enabled to compare relative change in entropy and information redundancy and to check the validity of the special case.

### 3.7. Defining Iso-Information Transmission and Iso-Information Redundancy Maps

Iso-information transmission is defined as the same proportion of information transfer capability that characterise two or more areas in a basin, between two hydrological phases. Hence it relates to the same level of gain or loss of information between phases in such specific areas respectively. Iso-information redundancy defines the same level of redundant information displayed by two or more areas in a basin, when the actual entropy of such areas is compared to their maximum theoretical entropy, for a specific phase.

The spatial representation of RCE gave the determination of iso-information transmission maps for WR90-WR2005 and WR2005-WR2012. Hence the zones of the same RCE could be determined and analysed. Similarly, the iso-information redundancy maps of TCs in the Middle Vaal was determined and analysed for WR90, WR2005 and WR2012. There is no universal rule to define entropic zone values; hence they are usually determined arbitrarily as far as entropy related studies are concerned [5,13,44]. For that, the range of RCE (*R*) was arbitrarily divided into intervals to define zones of the same information transmission (information redundancy). The maps in this case could show the level of dispersion or concentration of MAR over TCs, and be used as an indicator to monitor catchment yield (MAR) distribution in terms of water resources.

## 4. Results and Discussion

### 4.1. MAR Information Transmission in the Middle Vaal Basin between 1990 (2005) and 2005 (2012)

The computation of information transmission values between different phases presented in Table 2, relate to RCE values. It was found out that positive or negative values occurred. Positive values of information transmission mean loss while negative values yield gain. It can be observed that the values in this table are relatively small and vary between −0.067 to 0.038, i.e., −6.7% and 3.8% respectively. This could mean that no new major hydrological changes with respect to water resources occurred between the different phases in the Middle Vaal basin. Similar results were obtained in the case of the Upper Vaal Catchment [15]. This could also support that the framework of water resources management and development in the Middle Vaal region hasn’t undergone drastic changes; nonetheless there has been an update in the water strategy at national level. In fact the new national water strategy espouses fundamentally water management principles [45] similar to the old one.

It was also noticed that the values of information transmission derived for WR90-WR2005 and those for WR2005-WR2012 did not correlate significantly and the coefficient of correlation was −0.3. Nonetheless this could mean that, given a specific TC, MAR information transmission for WR90-WR2005 and WR2005-WR2012 varied slightly in opposite direction.

**Figure 2:**
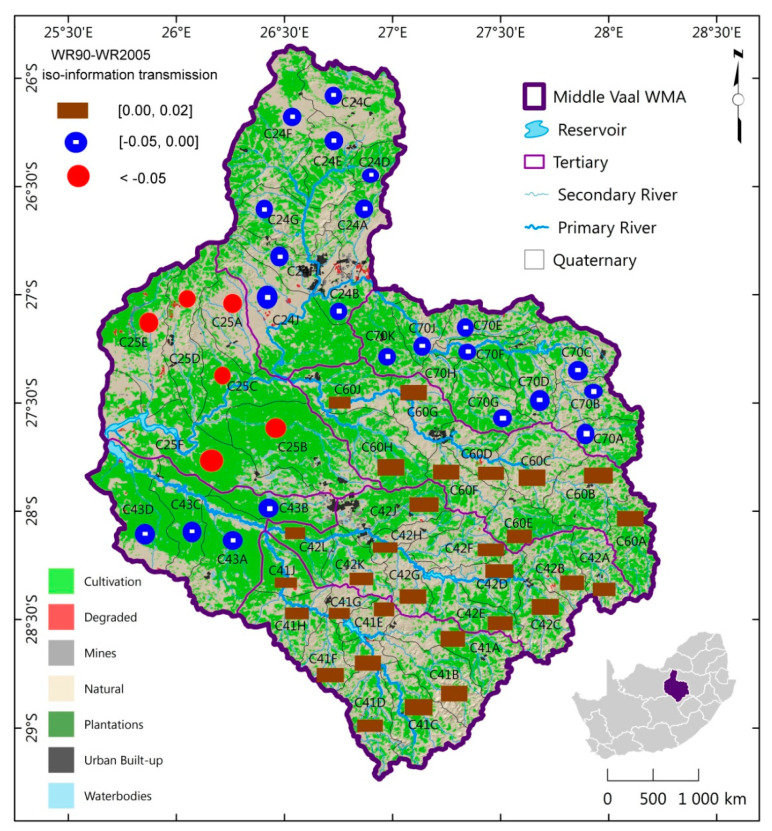
Spatial distribution of iso-information transmission for WR90-WR2005 in TCs (tertiary catchments) of the Middle Vaal basin or water management area (WMA).

**Figure 3:**
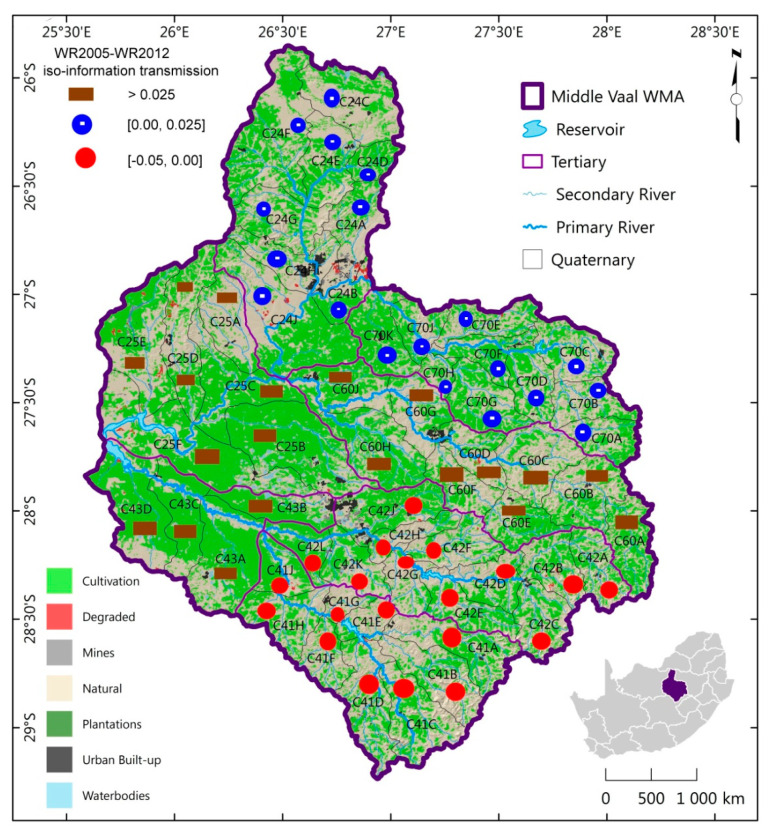
Spatial distribution of iso-information transmission for WR2005-WR2012 in TCs of the Middle Vaal basin.

The spatial distribution of iso-information transmission between the different phases was generated as shown in Figure 2 and Figure 3. 

These are maps for TCs of the same information gain or loss associated with MAR, between phases. For instance TCs C60, C41and C42 could be considered to have the same level of hydrological information transmission between WR90 and WR2005. A similar observation was made between WR2005 and WR2012. In particular, Figure 2 showed that the East South had the highest information loss while gain was concentrated more in East Northern part of the Middle Vaal for WR90-WR2005. Between 1990 and 2005, East South has moved towards the highest degree of uncertainty of MAR as opposed to East North.

Figure 3 showed that information gain was concentrated in the South part, i.e., C42 and C41 for WR2005 and WR2012; hence information loss occurred in the rest of the Middle Vaal basin. The highest loss of information was in the Central part with dominance towards the East part. It is believed that areas of highest information loss could be an alarming situation for water managers, especially when hydrological extreme events had to occur in those areas. It was observed that C43 and C70 was the only TC pair to have the same sign of information transmission, i.e., positive and negative for WR90-WR2005 and WR2005-WR2012 respectively.

### 4.2. MAR Information Redundancy in the Middle Vaal

The information redundancy values were computed from relative entropy values, as presented in Table 2. This table does include relative entropy since they were just used to derive redundancy. Table 2 shows also that information redundancy varied between 0.041 and 0.104, i.e., 4% and 10% respectively. The low values of information redundancy could mean that the uncertainty of MAR contained in each TC for the different phases was generally relatively higher. This could suggest that the MAR spatial distribution was associated with high dispersion or spread in the TCs of the Middle Vaal basin. For each phase, the small values of redundancy, i.e., high complexity ratio suggested that generally the MAR of TCs in the Middle Vaal were not concentrated in one or few areas.

High complexity could mean that more information could be needed to reduce the uncertainty associated with MAR of TCs in Middle Vaal. Enhancing good decision making would be needed for further development and operation of the water resources in this basin and could likely lessen uncertainty.

**Figure 4:**
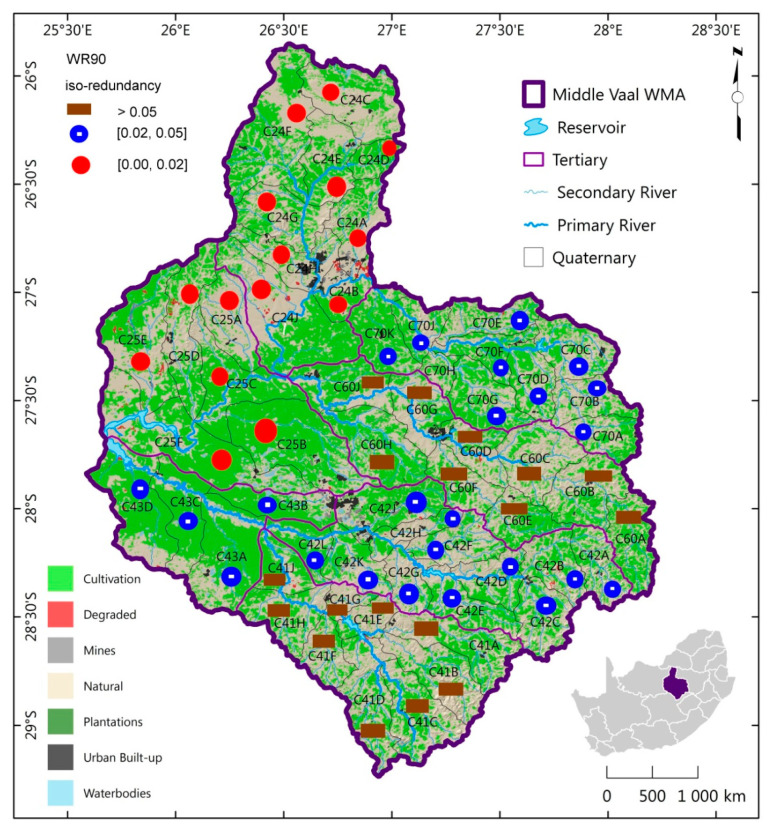
Spatial distribution of iso-information redundancy of TCs for WR90 in the Middle Vaal basin.

**Figure 5:**
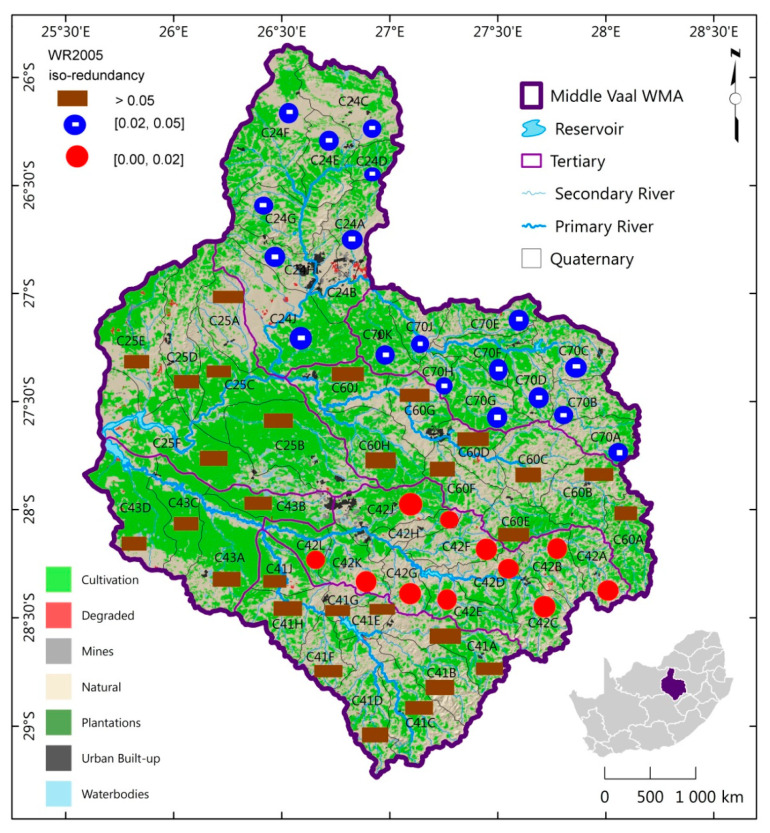
Spatial distribution of iso-information redundancy of TCs for WR2005 in the Middle Vaal basin.

**Figure 6:**
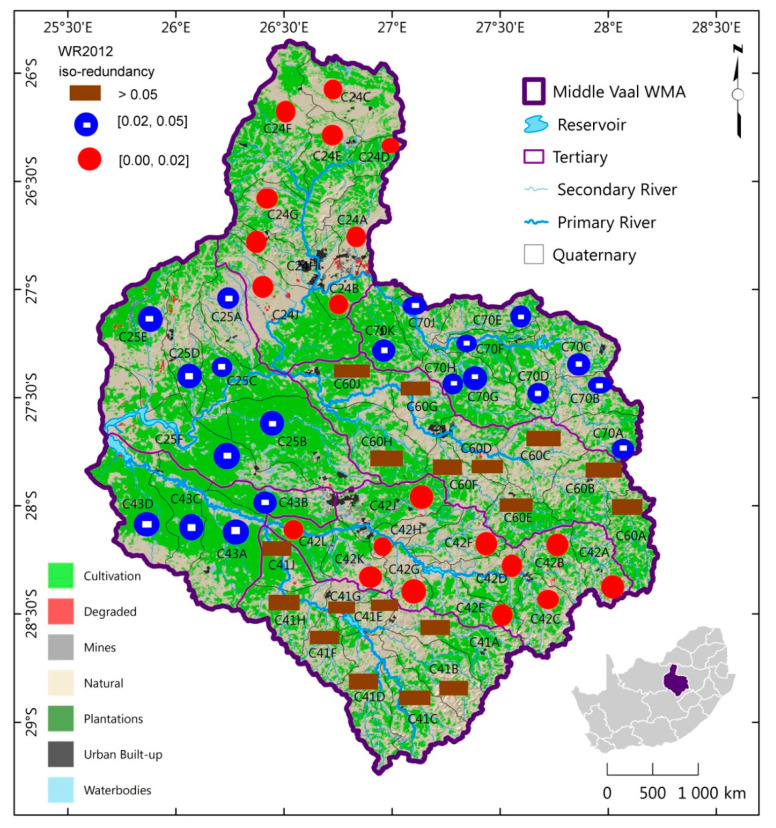
Spatial distribution of iso-information redundancy of TCs for WR2012 in the Middle Vaal basin.

The MAR spatial distribution of TCs of the same information complexity for each hydrological phase was generated as shown in Figure 4, Figure 5 and Figure 6. These figures depict the iso-information redundancy of MAR in the TCs of Middle Vaal for WR90, WR2005 and WR2012 respectively. Although its values were relatively small, the information redundancy was divided into 3 zones as displayed in the above-mentioned figures. For the 3 phases, the spatial distribution of information redundancy did not present exactly similar patterns.

For instance, in Figure 4, it could be observed that TCs C24 and C25 needed the same amount of information to reduce their uncertainty associated with MAR; similarly, for TCs C60 and C41; and C70 and C43. The first and last zones contained the same number of TCs, while more TCs were in the second zone. The zone of relatively higher complexity included tertiary catchments C24 and C25, which were situated mostly in the North West of Middle Vaal. The South East part had relatively high information redundancy, which corresponded to relatively low information complexity. The North West of the Middle Vaal could be believed to have relatively higher dispersed distribution of MAR than the South East. In general, due to small values of information redundancy in the different zones, the distribution of MAR was well dispersed across Middle Vaal for WR90.

In Figure 5, the zone of relatively high redundancy dominated WR2005 phase and covered the Central and Southern parts of the basin; i.e., C60, C41, C43 and C25. These parts could be believed to contain lower information associated with MAR as compared to the rest of the basin. TC C42 situated in East Southward displayed the lowest information redundancy followed by C24 and C70. C42 had relatively the highest complexity and presented the most likely dispersed spatial distribution of MAR. The West Southern and Central Eastern parts were dominated by relatively high information redundancy. Hence their complexity associated with MAR was relatively lower. Unlike WR90, the distribution of MAR was fairly spread across Middle Vaal for WR2005.

In Figure 6, the spatial distribution of MAR was depicted for WR2012. The North Western and North Eastern parts of Middle Vaal basin presented relatively low information redundancy, which were believed to be more complex than other parts.

For both WR2005 and WR2012, the spatial distribution of information redundancy had a similar pattern in South-East, i.e., C70, C60, C42 and C41. Although all 3 phases WR90, W2005 and W2012 had low level of information redundancy, their bottom Southern part, i.e., TC C41 had a high level of information redundancy, hence associated with relatively low level of complexity.

In general, it could be suggested that the existing information contained in MAR did not contain much of unnecessary repetition with respect to its spatial distribution in Middle Vaal. This aspect could be useful for efficiently developing, managing and operating water resources in this basin. However, entropy theory does not specify exactly the magnitude of MAR that constitutes the redundant information spatially and how it should be explored without impacting on the efficiency of water resources management. Nonetheless, in the current situation of water resources of South Africa, on a spatial distribution the overall picture at tertiary level could be that the information contained in MAR tends to be high while the level of redundancy seems to be less.

### 4.3. Comparison between Relative Change in Entropy and Information Redundancy of MAR

From the above results, C24 and C25 were the only TCs for which RCE and *R* values almost coincide in magnitudes. For these specific TCs, the information transmission magnitude for WR90-WR2005 could be seen as the equivalent of redundant information in WR2005. Therefore, for C24 and C25 situated in the North-East, the knowledge of redundant information at WR2005 alone could be sufficient to characterise the information transmitted between WR90 and WR2005.

This preliminary study was restricted only to entropy of MAR; however the inclusion of other variables (such as economic indicators, population growth, evaporation, rainfall, land use/change and many others) could affect the findings.

### 4.4. Hydrological Implications of Iso-Information Transmission/Redundancy

Referring to the previous section, for a specific tertiary catchment in a basin, iso-information transmission can be translated into areas that display the same level of information gain or loss between hydrological phases. For instance, loss of hydrological information in such areas would imply increase in uncertainty, thus increase in entropy of the MAR spatial distribution. Beyond the zone of catchment resilience, an increase of entropy in those specific areas could be exacerbated by the occurrence of extreme hydrological events such as floods or droughts [15]. These events could be associated with high costs, e.g., loss in human life, livestock and/or damage of water infrastructure. Hence high entropy of MAR associated with high information complexity could be a warning sign for the management and operation of water resources, which could be vulnerable. In this way, decision-makers could use iso-information transmission maps in identifying likely areas of the same level of vulnerability, resilience or sustainability in so far as water resources are concerned.

For areas in a tertiary catchment within the same hydrological phase, iso-information redundancy depicts the same degree of information complexity associated with MAR. High information complexity could show a high dispersion of MAR in areas of TCs, hence associated with relatively low redundancy. Previous studies showed that the determination of the minimum redundant information was critical for optimizing hydrological networks [27,29]. In this respect, hydrologists made use of adequate information by reducing the minimum information redundancy contained in different hydrological stations/gauges. Hence water managers could use iso-information redundancy maps to determine areas depicting a certain proportion of unnecessary information. This information could not be of use and could probably be costly in practice, e.g., for design, operation and optimisation of water resources. From a theoretical aspect, threshold values for iso-information transmission and iso-information redundancy remain still to be determined, with respect to MAR as a unique variable. The determination of thresholds could be convoluted when additional climatic variables and anthropogenic factors could be considered, besides runoff.

## 5. Conclusions

The development of the Middle Vaal basin catchment is instrumental to Gauteng, which is the economic hub of South Africa. The information redundancy contained in MAR data as the equivalent information complexity was evaluated to assess the level of dispersion of MAR in the basin. Based on WR90, WR2005 and WR2012 datasets, the MARs in the TCs of Middle Vaal basin were characterised by low redundancy (high complexity), hence they could contain useful information for further development and management of water resources in the basin.

The study has also the merit to have derived the spatial iso-information transmission and iso-information redundancy distribution of TCs between 1990 and 2012. The distributions of MAR illustrated in the form of maps could be useful for water resources managers and planners to better understand the evolution of MAR and monitor the catchment yield in the form of runoff. Information transmission was translated into highest degree of uncertainty associated with MAR in the East Southern part of the Middle Vaal between 1990 and 2005; and in the Eastern part between 2005 and 2012. Water resources decision-makers should deal with care the situation of high entropy, when approaching extreme events such as droughts and floods.

An assessment of information redundancy revealed that the proportion of relatively low complexity was prevalent in the South part of the Middle Vaal for all 3 datasets. The pattern of spatial distribution of information redundancy was not strictly the same everywhere; nonetheless there was partially similarity between WR2005 and WR2012, specifically for the East South part of the basin. In the South of Middle Vaal, the information complexity was relatively low to be likely associated with low dispersed MAR in TCs. Generally information transmission and information redundancy were relatively small and could not exceed 10%.

In a very specific case, it could be possible to determine directly information transmission between 2 phases from the information redundancy for only one phase. Although information redundancy was shown to be minimal through the current state of water resources management in the Middle Vaal, theoretic entropy concept does not give the magnitude of MAR that contributes to redundant information and how this should be used or removed in order to maintain an acceptable level of efficiency for managing water resources.

This preliminary study has the limitation of defining spatial complexity based on MAR only. Further research could investigate the impact of hydrological variables (other than runoff) and anthropogenic factors on the spatial distribution of information redundancy. The evaluation of complexity in this multivariate configuration could be enlightening. It could be suggested that the study be expanded to secondary catchments in the Middle Vaal and to basins other than Middle Vaal. Updated publications beyond WR2012 data sets could be used when available.

## Figures and Tables

**Table 1:** Depicts tertiary catchments including their number of quaternary (QCs), catchment area, MAR (mean annual runoff), MAE (mean annual evaporation) and MAP (mean annual precipitation) in the Middle Vaal basin, as obtained from [37].

Tertiary Catchment	Catchment Area (km^2^)	MAE (mm)	MAP (mm)	MAR (WR90) × 10^6^ m^3^	MAR (WR2005) × 10^6^ m^3^	MAR (WR2012) × 10^6^ m^3^	Number of QCs
C24	7512	1291	418	174	154	153	9
C25	7055	1475	418	36	27	37	6
C41	6994	1431	514	317	199	193	9
C42	7555	1418	618	226	197	181	11
C43	2765	1119	306	11	10	22	4
C60	6765	1352	503	166	178	178	9
C70	6157	1496	555	192	147	155	10

**Table 2:** Shows entropic calculations, i.e., marginal entropy in decibels (dB), information transmission and redundancy for tertiary catchments (TC) in the Middle Vaal basin.

Tertiary Catchment (TC)	Entropy (S) (dB)			Information Transmission (RCE)		Redundancy (*R*)		
	WR90	WR2005	WR2012	WR90-WR2005	WR2005-WR2012	WR90	WR2005	WR2012
C24	0.899	0.870	0.888	−0.033	0.022	0.0041	0.037	0.016
C25	0.776	0.724	0.745	−0.067	0.029	0.0022	0.069	0.042
C41	0.884	0.889	0.873	0.0060	−0.018	0.073	0.068	0.084
C42	1.020	1.025	1.024	0.0045	−0.0011	0.020	0.015	0.016
C43	0.584	0.565	0.587	−0.003	0.038	0.03	0.061	0.026
C60	0.854	0.870	0.892	0.018	0.026	0.104	0.088	0.064
C70	0.974	0.970	0.971	−0.0042	0.0010	0.026	0.030	0.029

RCE and *R* are defined on a unit scale, and can be converted into % by multiplying by 100.
